# Study on Genetic Variance of miR-541 in Type 1 Diabetes

**DOI:** 10.5402/2012/630861

**Published:** 2012-12-11

**Authors:** Bei Han, Xing Shi, Quan Peng, Wentao Gao

**Affiliations:** ^1^Department of Endocrinology, Nanjing Children's Hospital Affiliated to Nanjing Medical University, Nanjing 210029, China; ^2^Division of Biliary and Pancreatic Disease, Department of General Surgery, The 1st Affiliated Hospital of Nanjing Medical University, No. 300 Guangzhou Road, Nanjing 210029, China

## Abstract

Genetic susceptibility plays a key role in type 1 diabetes development. Because miR-541 gene was located within the associated chromosome loci and its target genes include the diabetes-associated gene neurogenin3, this study aimed to investigate whether miR-541 had type 1 diabetes-associated genetic variations. Type 1 diabetes children and healthy volunteers were recruited; direct sequencing was performed in initial 69 patients and 46 volunteers. We identified 1 reported SNP (rs12893725) and 3 novel genetic variations, for the candidate -404 G→T variation, restriction fragment length polymorphism (RFLP) was performed in total 247 diabetes children and 212 healthy volunteers, a different distribution trait of allele frequencies was found between the two groups, and further clinical analysis found no significant correlation between clinical parameter and genotypes among patients. In addition, by luciferase reporter assay, -404 was found to be within putative promoter region of pre-miR-541; although mutation of G→T has no effect on promoter activity, a significant secondary structure alteration may possibly influence its processing and transcription. In conclusion, we identified 3 novel genetic variations in putative promoter of miR-541 in type 1 diabetes patients; -404 G→T of miR-541 is a potential T1D-associated genetic variation.

## 1. Introduction

Patients with type 1 diabetes are likely to carry strong genetic predispositions [1, 2]. A group of insulin-dependent diabetes mellitus (IDDM) susceptibility loci, including 19 IDDM loci on human chromosomes, have been identified [3].

MicroRNAs (miRNAs) play an important role in the posttranscriptional regulation mechanism in eukaryotic cells [4]. It has been reported that miRNAs are associated with the development of pancreatic *β* cells, production and secretion of insulin, and insulin action on target organs, such as adipose tissue, liver, and skeletal muscle [5–10] and, more importantly, in immune regulation and autoimmune diseases [11], it can be speculated that miRNAs might affect the development of type 1 diabetes which was characterized as autoimmune insulitis with genetic susceptibility. Indeed, a miRNA expression profile has been reported for type 1 diabetes cell model recently [12].

Several reports on the genetic variations, single nucleotide polymorphisms (SNPs), and mutations in miRNAs have been reported in genetic diseases and tumor somatic and germ cells [13, 14], which can lead to dysfunctional regulation of miRNAs [15, 16]. Bioinformatic analysis has led to the identification of many miRNAs in the IDDM region of the type 1 diabetes-susceptible chromosome [17]; miR-541 is located at 14q32 in the IDDM16 region and plays a crucial role in the development of pancreas [18]. Its target genes include diabetes-related gene neurogenin3 (NGN3) [19]. The present study focuses on the genetic variants of the miRNA miR-541 to investigate its association with type 1 diabetes.

## 2. Materials and Methods

### 2.1. Patient Group

Total 247 children diagnosed with type 1 diabetes between January 2006 and April 2012 at the Department of Endocrinology, Nanjing Medical University Associated Nanjing Children Hospital were selected. Initially, 69 children were selected for sequencing to screen; then all patients were included for secondary restriction fragment length polymorphism (RFLP) study for identified genetic variance. Sequencing group includes 36 females and 33 males, with an average age of 11.02 ± 2.48 years; RFLP group includes 119 females and 128 males, with average age of 7.52 ± 4.06 years. The diagnosis was made on the basis of the criteria approved by the Chinese Diabetes Association in 1999.

Total 212 healthy volunteers were selected as controls, including 118 female and 94 male, with an average age of 9.21 ± 4.32 years; the initial 46 controls for sequencing include 24 females and 22 males (average age, 11.12 ± 2.59 years). All patients and volunteers provided written informed consent, and the study protocol was approved by ethics committee of Nanjing Children Hospital.

Peripheral anticoagulant plasma was collected. Ultrapure Genomic DNA Fast-Extraction kit was used to extract DNA that was stored at −20°C until further use.

### 2.2. Study Area and Polymerase Chain Reaction Amplification of Pre-miR-541

2 overlapping primers were designed, with an amplification area in the −1084 to +167 bp region of the pre-miR-541, so that its promoter (regulation) region and pre-miRNA were also included. The sequence of the primers used is shown in Table 1. Polymerase chain reaction (PCR) protocol was used as follows: denaturation at 95°C for 5 min, elongation at 55°C, 35 cycles, followed by 72°C for 5 min. GoTaq DNA polymerase used for PCR amplification was purchased from Promega Corporation. 

### 2.3. DNA Sequencing

ABI3730 sequencer (Gene Company) was used for sequencing analysis. Both forward and reverse sequencing reactions were performed. 

### 2.4. PCR-Restriction Fragment Length Polymorphism (RFLP)

For the variation point -404 G→T identified by direct sequencing, RFLP analysis was performed using -404 G→T-specific restriction enzyme *Dpn*II.

### 2.5. Luciferase Reporter Gene Assay

Design primers containing restriction enzyme sites EcoRI and HindIII, MiR541_P_EcoRI-F: CG-GAATTC-GCGTTTCTCATGAGCCTTTC, MiR541_P_HindIII-R: TCCG-AAGCTT-CAACC TTCCC CAGAC TCAGA, the amplified fragment is from −780 bp ~ +167 of the pre-miR-541, wild type and -404 G→T mutated fragments were constructed into PGL3 BASIC, respectively. Validate these plasmids through sequencing in INVITROGEN. Set PRL as internal control. Collect the lysates after 48 h and measure fluorescence (integration: 10 s). 

The influence of genetic mutation on the secondary structure of miR-541 precursor RNAfold web server was used to predict the secondary structure of the wildtype and -404 G→T mutated miR-541.

### 2.6. Data Analysis

Comparisons of genotype frequencies between the groups were conducted using a chi-square test; comparisons of clinical parameter were conducted using Student's *t*-test (SAS software). A *P* value of <0.05 was considered significant.

## 3. Results

### 3.1. A Known SNP (Reported in the SNP Database) and 3 Novel Genetic Mutations Were Identified by DNA Sequencing

**Figure 1:**
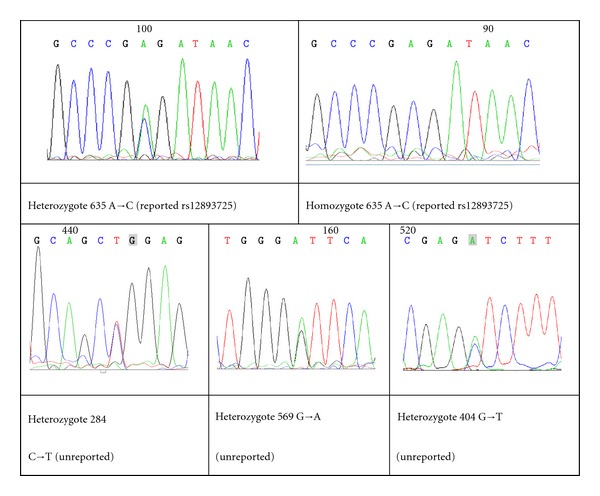
A known SNP and 3 novel genetic mutations were identified by DNA sequencing. In this study, 1 reported single nucleotide polymorphism (SNP) (rs12893725) (in SNP database) and 3 unreported gene mutations were identified by sequencing. Heterozygote -284 C→T and heterozygote -569 G→A were newly identified SNPs. Heterozygote -404 G→T was observed in only 3 diabetic patients.

Sequencing study includes 69 T1D patients and 46 controls. By direct sequencing, 1 reported single nucleotide polymorphism (SNP) (rs12893725) (in SNP database) and 3 unreported gene mutations were identified, including heterozygote -284 C→T, heterozygote 569 G→A and heterozygote -404 G→T (Figure 1).

The -635 homozygote and heterozygote variant rs12893725 reported in the SNP database were detect with high frequency in the type 1 diabetes (27/69 and 12/69) and normal children (16/46 and 4/46); no significant difference was observed between the diabetes and normal children. Newly identified heterozygote polymorphisms -284 C→T were observed in both patients (11/69) and controls (5/46), with no statistical difference. Newly identified heterozygote polymorphisms -569 G→A were also observed in both patients (11/69) and controls (7/46), with no statistical difference (Table 2). 

Heterozygote -404 G→T was observed in only 3 diabetic patients but not in the first group of 46 controls, with statistical significance.

### 3.2. RFLP Analysis of miR-541 -404 G→T Reveals a Different Distribution of Allele Frequencies between Type 1 Diabetes Patients and Healthy Volunteers

**Figure 2:**
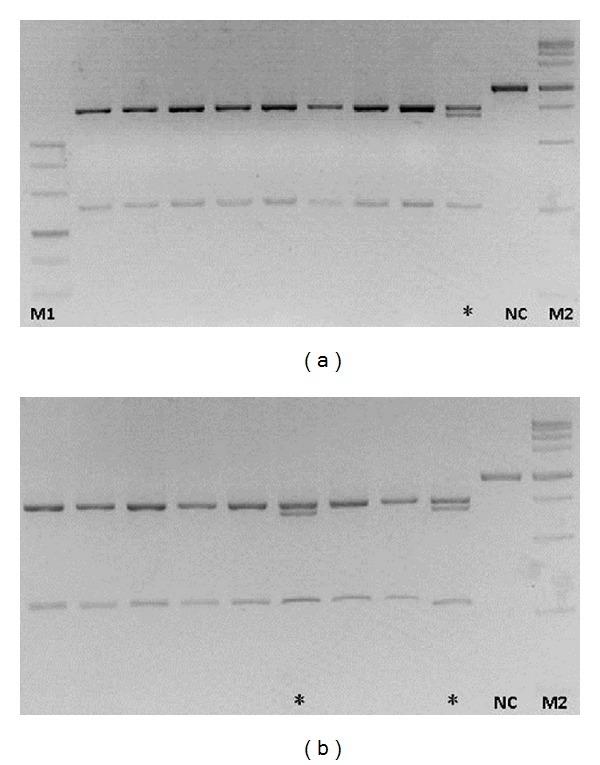
Restriction fragment length polymorphism (RFLP) analysis for -404 G→T. Polymerase chain reaction (PCR) amplification length was 992 bp. Nonmutated amplification region includes 1 *Dpn*II site, generating 741 + 251 digested fragments. However, for heterozygote -404 G→T mutation, another *Dpn*II site (GAGC→GATC) is present, thereby generating 4 digested fragments 741 + 680 + 251 + 61 (the 61 bp fragment was extremely small and hence could not be visualized). NC is the negative control (without enzymatic digestion), M1 and M2 for marker, *for samples with heterozygote -404 G→T mutation.

RFLP screening was performed for the heterotype -404 G→T mutation using specific *Dpn*II restriction enzyme in 247 type 1 diabetes patients and 212 healthy volunteers. Primer 2 that was 992 bp long was used for the RFLP analysis. -404 G→T polymorphism was observed in 9/247 patients and 4/212 healthy volunteers by RFLP, indicating that it was a rare SNP (Figure 2). 

The genotype frequencies of GG, GT, and TT were 96.36%, 3.24%, and 0.40% in type 1 diabetes patients and 98.11%, 1.89% and 0% in healthy volunteers, respectively. The frequencies of G and T allele were 97.98% and 2.02% in type 1 diabetes patients and 99.06% and 0.94% in healthy volunteers (Table 3); the allele frequencies are significantly different between diabetes patients and healthy controls.

### 3.3. No Significant Correlation between Clinical Parameter and Genotype of miR-541 -404 G→T in Type 1 Diabetes Children

Clinical parameters of type 1 diabetes children are analyzed, including sex, age, weight, C peptide (0 min and 120 min), HbAC (glycosylated hemoglobin), and ketoacidosis (Table 4). Compared to wildtype GG, there was no significant difference for genotype GTs in all clinical parameters, although GT have a tendency of lower serum C peptide, but all *P* values >0.05. 

### 3.4. Luciferase Reporter Gene Assay Shows −780 bp ~ + 167 Region of miR-541 Containing Promoter Activity; However miR-541 -404 G→T Had No Significant Influence on Promoter Activity

From −780 bp ~ +167 of the pre-miR-541, wildtype and -404 G→T mutated fragments were constructed into PGL3 BASIC, respectively. In contrast to PGL3 BASIC control vector, the constructed plasmid containing −780 bp ~ +167 regions had promoter activity, indicating a putative promoter of miR-541 in this region. 

**Figure 3:**
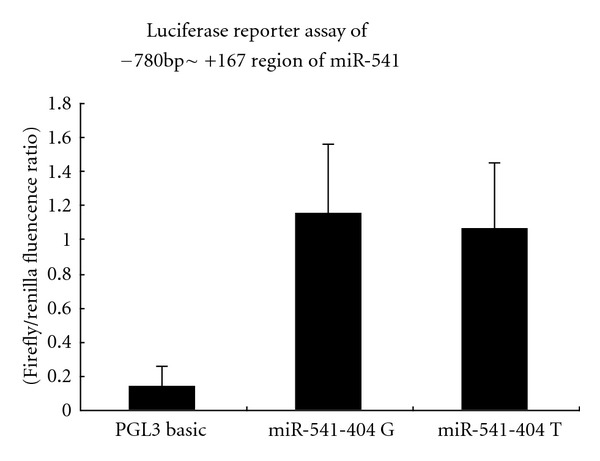
Luciferase reporter assay of −780 ~ +187 region of miR-541. In contrast to PGL3 BASIC control vector, the constructed plasmid containing −780 bp ~ +167 regions had promoter activity, indicating a putative promoter of miR-541 in this region. However, there is no significant difference in promoter activity between wildtype and -404 G→T mutation construct. *P* < 0.05 compared with PGL3 BASIC control.

However, there is no significant difference in promoter activity between wildtype and -404 G→T mutation construct (Figure 3). 

### 3.5. Mutation in -404 G→T Altered the Secondary Structure of miR-541 Precursor, which May Influence the miR-541 Processing and Maturation

**Figure 4:**
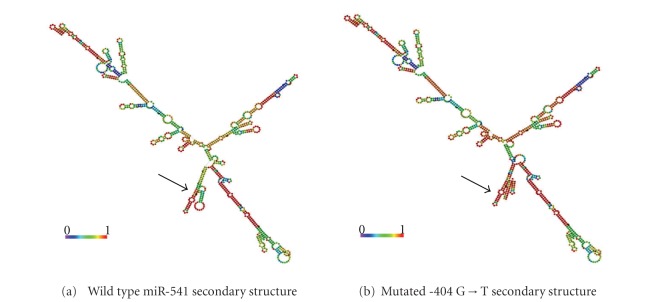
Alteration in the genetic sequence alters the secondary structure of the miR-541 precursor. The RNA secondary structure predicted by RNAfold web server indicated that a change in the secondary structure of miR-541 precursor may alter the base pair match of its precursor root region. Black arrows indicate the change in the sequence and secondary structure. The most stable secondary structure was selected on the basis of minimum free energy (MFE).

It is essential that the miRNA precursors have an appropriate secondary structure and *cis*-element during the precursor processing and maturation stages. In our study, although most of the mutations did not cause significant alterations in the secondary structure of miRNAs, the -404 G→T mutation altered the secondary structure of pre-miR-541 at the ring-shaped region, which is adjacent to the root ring structure of pre-miR-541, which may influence the miR-541 processing and maturation (Figure 4).

## 4. Discussion

This study found one reported SNP and 3 unreported SNPs for the first time. Among them, -404 G→T was found to be a rare SNP; a different distribution trait of allele frequencies of -404 G→T was found between type 1 diabetes patients and healthy volunteers, -404 located within promoter of pre-miR-541; although G→T has no effects on promoter activity, it leads to a significant secondary structure alteration of pre-miR-541, which may possibly influence its processing and maturation. 

Newly identified heterozygote -404 G→T was a rare SNP associated with type 1 diabetes; a different distribution trait of allele frequencies of -404 G→T was found between type 1 diabetes patients and healthy volunteers. By comparison of biochemical characterization and clinical parameters between different -404 alleles among patients, we found a lower c-peptide value (both 0 and 120 min) in T allele compared with wildtype G allele; however no statistic significance was found. Since it is a rare SNP and a relative low incidence of type 1 diabetes, it is hardly to significantly increase sample size in a single center, so a further multicenter analysis may be necessary to validate its clinical significance. 

-404 located within putative promoter of pre-miR-541 identified by luciferase reporter assay. Current study shows G→T variant has no effects on promoter activity in luciferase report assay. By RNAfold prediction, -404 G→T variant leads to a significant secondary structure alteration of pri-miR-541, which may possibly influence its processing and maturation.

If the -404 SNP affects transcription or function of miR-541 and this miRNA in turn may modulate target genes like neurogenin3, it would be desirable to include measurements related to mRNA or protein levels for this or other pertinent genes that may be affected by the 404 GT SNP variant. However, miR-541 is reported to be expressed in pancreas and contribute to pancreas development and regeneration [18]; it is generally impossible to get tissue samples from patient, while study from peripheral blood would be of little value compared with study in pancreas and target organ. Further in vitro study with cell line and animal model would be our next plan, to validate the biologic value of miR-541 and its -404 SNP. Interestingly, recent study shows that dysregulation of miR-541 in skin contributes to diabetic wound healing in mice model [20], and miR-541 in neuron contributes to neurite outgrowth in rat model [21], so an expression profile in blood and available target tissue such as skin and nerve in human are desirable.

## Figures and Tables

**Table 1:** Primers for mir-541 and replication region.

	Primer 1	Primer 2
Replication region	−780~+167	−1084~−112
Replication length	947 bp	992 bp
5′ primer	GCGTTTCTCATGAG	TCAGTGGGGTCTGGTCTTTC
3′ primer	CAACCTTCCCCAGA	CAGACGACTTCCCTTCAGG

**Table 2:** Comparison of mir-541 genetic variation identified by direct sequencing between type 1 diabetes children and control groups.

	Type 1 diabetes patients *n* = 69	Healthy controls *n* = 46
Homozygote variant -635 rs12893725	27 (39.13%)	16 (34.78%)
Heterozygote variant -635 rs12893725	12 (17.39%)	4 (8.70%)
Heterozygote -284 C→T	11 (15.94%)	5 (10.87%)
Heterozygote -569 G→A	11 (15.94%)	7 (15.22%)
Heterozygote -404 G→T	3 (4.35%)*	0

*Comparison with control group, *P* value < 0.05.

**Table 3:** Comparison of genotype and allele frequency of mir-541 -404 G→T identified by RFLP between type 1 diabetes children and control groups.

		Genotype frequency						Allele frequency*			
		GG		GT		TT		G		T	
	*n*	*n*	%	*n*	%	*n*	%	*n*	%	*n*	%
T1D	247	238	96.36	8	3.24	1	0.4	484	97.98	10	2.02
Control	212	208	98.11	4	1.89	0	0	420	99.06	4	0.94

*P < 0.05.

**Table 4:** No significant correlation was found between clinical parameter and allele of mir-541 -404 G→T in type 1 diabetes children.

Allele	*n*	Sex male/female	Average age (year)	Average weight (kg)	C-peptide 0′	C-peptide 120′	HbAC %	Ketoacidosis
G	238	124/114	7.49 ± 4.04	23.92 ± 11.18	0.17 ± 0.16	0.53 ± 0.65	12.19	78/238
T	9	4/5	7.43 ± 4.84	22.23 ± 10.86	0.16 ± 0.12	0.32 ± 0.19	12.37	4/9
